# Concurrent-Validity and Reliability of Photocells in Sport: A Systematic Review

**DOI:** 10.5114/jhk/174285

**Published:** 2023-11-28

**Authors:** Weilhelmn Multhuaptff, Eneko Fernández-Peña, Adrián Moreno-Villanueva, Alejandro Soler-López, Markel Rico-González, Filipe Manuel Clemente, Sergio Bravo-Cucci, José Pino-Ortega

**Affiliations:** 1 Facultad de Ciencias de la Salud, Universidad Peruana de Ciencias Aplicadas – UPC, Lima, Perú.; 2 Department of Physical Education and Sport, University of the Basque Country UPV/EHU, Vitoria-Gasteiz, Spain.; 3 Faculty of Health Sciences, Isabel I University, Burgos, Spain.; 4 BIOVETMED & SPORTSCI Research group, Faculty of Sports Sciences, University of Murcia, San Javier, Spain.; 5 Faculty of Sports Sciences, University of Murcia, San Javier, Spain.; 6 Department of Didactics of Musical, Plastic and Corporal Expression, University of the Basque Country UPV/EHU, Leioa, Spain.; 7 Escola Superior Desporto e Lazer, Instituto Politécnico de Viana do Castelo, Viana do Castelo, Portugal.; 8 Department of Biomechanics and Sport Engineering, Gdansk University of Physical Education and Sport, Gdansk, Poland.; 9 School of Rehabilitation Therapies, Faculty of Medical Technology, Federico Villarreal National University, Lima, Peru.

**Keywords:** sports technology, accuracy, repeatability, timing in sport

## Abstract

Specific physical qualities such as sprint running, change-of-direction or jump height are determinants of sports performance. Photocell systems are practical and easy to use systems to assess the time from point A to point B. In addition, these photoelectric systems are also used to obtain the time of vertically displaced movements. Knowing the accuracy and precision of photocell timing can be a determinant of ensuring a higher quality interpretation of results and of selecting the most appropriate devices for specific objectives. This systematic review aimed to identify and summarize studies that have examined the validity and reliability of photocells in sport sciences. A systematic review of PubMed, SPORTDiscus, and Web of Science databases was performed according to the Preferred Reporting Items for Systematic Reviews and Meta-Analyses (PRISMA) guidelines. From the 164 studies initially identified, 16 were fully reviewed, and their outcome measures were extracted and analyzed. Photocells appear to have a strong agreement with force plates (gold standard), but are not interchangeable to measure the vertical jump. For monitoring horizontal displacement, double beam systems, compared to single beam systems, are more valid and reliable when it comes to avoiding false triggers caused by swinging arms or legs.

## Introduction

Specific physical qualities such as sprinting, change-of-direction (COD) or jump height are determinants of sports performance (Faude et al., 2012; Haugen et al., 2014a; Soler-López et al., 2022). These capacities lead to the ability to reach a specific target in the fastest time possible in linear, curvilinear, or both types of trajectories with or without COD, as well as an athlete's ability to reach great vertical jump heights (Haugen et al., 2014a; Soler-López et al., 2022). As determinants of sports performance, sprinting, COD ability and jump height are part of periodically applied fitness assessment batteries (Soler-López et al., 2022; Willberg et al., 2023). While instantaneous speed is regularly assessed using a radar gun or video-based analysis (Bataller-Cervero et al., 2019; Romero-Franco et al., 2017; Uysal et al., 2023), sprinting time, COD time and jump height are usually measured using photocells.

Photocell systems which work by breaking a light beam are electronic devices that consist of a light emitting module and a receiving module sensitive to changes in brightness intensity; in many cases a mirror is also used to reflect the light beam to the receiving LED (Bond et al., 2017b; Haugen et al., 2014a; Haugen and Buchheit, 2016). Each time an object or subject cuts the beam, the system triggers a signal that activates and introduces a marker or stops the timer. Considering that photocells are practical and easy to use, their implementation to assess the time to cover a distance from point A to point B has become a regular practice for assessing sprinting and COD (Yeadon et al., 1999). In addition, to obtain the time of movements with vertical displacement, these photoelectric systems use an array or a matrix of emitting LEDs (between 10 and 96 LEDs), organized in two parallel bars (one emitting and one receiving) (Attia et al., 2017; García-López et al., 2013; Glatthorn et al., 2011).

There are different types of photocells depending on how they operate (Haugen and Buchheit, 2016): (i) single-beam; (ii) dual-beam; and (iii) split-beam and post-processing. A pair of single-beam photocells is characterized by the use of a transmitter emitting an infrared beam to a reflector (positioned directly opposite) that reflects the beam back to the transmitter (Haugen and Buchheit, 2016). This can be used for situations in which any part of the body can be used to trigger the photocell. Dual-beam photocells (also termed as double-beam) consist of positioning two photocells at different heights and both beams must be broken to work the trigger, thus it is useful to avoid specific situations of triggering with lifted knees or swinging arms (Haugen and Buchheit, 2016). Because they condition the requirements for triggering, dual-beam systems ensure greater accuracy and reliability (Yeadon et al., 1999), thus being more recommended for scientific research.

As an alternative to single or dual-beam, split-beam photocells consist of using the same infrared beam that is split by a metallic device which will emit this infrared beam in two reflectors interspaced vertically by 20 to 30 cm (Haugen and Buchheit, 2016). Both beams should also be broken to trigger the photocell. However, this process seems to produce greater noise than when using dual-beam photocells (Haugen et al., 2014b; McBride et al., 2002; Wong et al., 2010). Another approach can be to use post-processing timing systems in which software scans all signals and processes the information to remove false signals.

As mentioned before, the use of photocells may constrain the accuracy and precision of data collection (Haugen et al., 2014b). However, the device itself can be also a determinant of reducing the bias and ensuring proper conditions for assessing human performance (Enoksen et al., 2009). In fact, in any performance analysis, reducing the error of the device should be ensured, otherwise, the inference about performance can be erroneous, based on the error of the device and not the variability of human performance (Hopkins, 2000; Hopkins et al., 2009). Experimental conditions should also be considered since, for example, different starting positions and experimental procedures may affect the accuracy and precision of results (Haugen et al., 2012).

Therefore, knowing the accuracy and precision of photocell timing can be a determinant of ensuring a higher quality interpretation of results and of selecting the most appropriate devices for specific objectives. In spite of the relevance of the topic, there has been no systematic review that summarizes the evidence about concurrent validity and reliability of photocells for use in sports analysis. This can be decisive for helping coaches and sports scientists to identify the accuracy and precision levels of different models, brands, and experimental conditions. For this reason, the purpose of this systematic review was two-fold: (i) to summarize the evidence about the concurrent validity of photocell timing for sports analysis; and (ii) to summarize the evidence about the reliability of photocell timing for sports analysis. Particular attention was also paid to the applied experimental procedures.

## Methods

### Experimental Approach to the Problem

A systematic review was performed in accordance with PRISMA (Preferred Reporting Items for Systematic Reviews and Meta Analyses) guidelines for performing systematic reviews in sports science (Moher et al., 2015).

### Search Strategy

A systematic search of three databases (PubMed, Web of Sciences and SPORTDiscus) was performed to identify articles published prior to October 22, 2022. The PICO (Patient, Problem, or Population − Intervention or Exposure − Comparison, Control, or Comparator − Outcome/s) design was used to provide an explicit statement of the state of the question.

Priority was given to studies focused on the assessment of photocells. Three main groups of words were established: (1) population: “athletics”, “team sport”, “sprinter”; (2) intervention: “assessment”, “assessing”, “evaluation”, “method”, “protocols”, “test*”; (3) outcomes: “jump height”, “sprint time”, “contact time”, “running speed”. Words from different groups were combined to extract as many items as possible by adding Boolean markers. Clusters of keywords (population, intervention, and outcome) were connected with OR within each cluster and AND was used to combine the three groups: (athletics or “team sport” or sprinter) AND (assessment OR assessing OR evaluation OR method OR protocol* OR test) AND (“jump height” or “sprint time” or “contact time” or “running speed”). Additionally, the reference lists of the studies retrieved were manually searched to identify potentially eligible studies not captured by the electronic searches.

### Screening Strategy and Study Selection

When the referred authors had completed the search (M.R.-G., A.M.-V. and J.P.-O.), they compared the results among themselves to ensure that the same articles were identified. Then, one of the authors (M.R.-G) downloaded the main data from the articles (title, authors, journal, date and databases) and put them onto an Excel spreadsheet (Microsoft Excel, Microsoft, Redmond, USA). Then, two authors (A.M.-V. and J.P.-O.) removed duplicates. The remaining articles were screened and checked by two authors independently (A.M.-V. and J.P.-O.) against the inclusion and exclusion criteria (Table 1). There were discussions with a third author (A.S.-L.) in the event of discrepancies regarding the selection process. Possible errata for the included articles were considered. Moreover, relevant articles not previously identified were also screened in an identical manner and further studies that complied with the inclusion and exclusion criteria were included and labelled as ‘included from external sources’.

**Table 1:** Inclusion/exclusion criteria.

	Inclusion	Exclusion
Population	Tests were conducted in healthy athletes or recreationally healthy active adults (who were used to performing the test or technical gesture used in the intervention protocol of each study).	Tests were not conducted in athletes (e.g. pregnant women, elderly) or in healthy active adults (e.g. injury). No previous experience in performing the test or technical gesture used in the intervention protocol of each study.
Intervention	Concerned with the validity and/or reliability of commercially available photocells that monitor sprint time, run time, jump height, flight time and/or contact time.	Not concerned with validity or reliability of photocells, and/or assessing variables other than those mentioned in the inclusion criteria.
Comparator	Considering validity, photocells were compared to the recognised gold standard (high-speed cameras, 3D motion capture, force plates and/or photocells widely recognised by scientific literature).	For validity, photocells were not compared with recognised reference methods or with other photocells that are not widely used in sports.
Outcomes	Studies that describe speed variables (horizontal displacement photocells) and/or a vertical jump (vertical displacement photocells).	Studies that do not describe speed and/or a vertical jump.
	Considering validity, one of the following measures was included: (i) intraclass correlation coefficient; (ii) correlation coefficient; (iii) limit of agreement.	For validity, outcomes did not refer to intraclass correlation coefficients, correlation coefficients, standard error of estimate, nor minimal product regression.
	For reliability, one of the following measures was included: (i) intraclass correlation coefficient; (ii) typical error; (iii) coefficient of variation; (iv) standard error of measurement.	For reliability, outcomes did not refer to intraclass correlation coefficients, typical error of measurement, coefficients of variation, nor standard error of measurement.
Study	Original research and full-text studies.	Other article types than original (e.g., reviews, letters to editors, trial registrations, proposals for protocols, editorials, book chapters and conference abstracts).

### Data Analysis

The information collected from the selected studies addressed the following aspects: (1) anthropometric characteristics of the sample (sex, age, body mass, height); (2) characteristics of the assessment protocols (location of photocells, photocell technology, measurement variables, assessment test and tools used); (3) results obtained with the tests carried out; (4) conclusions endowed with scientific rigor and objectivity, which helped in the analysis of the casuistry of the data obtained.

### Quality of Studies

The methodological assessment process was performed by two authors (A.M.-V. and M.R.-G.) using an adapted version of the STROBE assessment criteria for cross-sectional research (O’Reilly et al., 2018), looking for studies that were eligible for inclusion. Each article was assessed based on 10 specific criteria (Table 2). Any disagreement was discussed and solved by consensus. Each item was evaluated using numerical characterization (1 = completed; 2 = non-completed). As suggested by O’Reilly et al. (2018), each study rating was qualitatively interpreted using the following law: the study has a risk of bias or low quality with a score lower or equal to 7 points, while those studies with higher scores are considered as of low risk of bias or high quality.

**Table 2:** Methodological assessment of the included studies.

Reference	1	2	3	4	5	6	7	8	9	10	Quality
Altmann et al. (2018a)	1	1	0	1	1	1	1	1	1	1	High
Altmann et al. (2018b)	1	1	0	1	1	1	1	1	1	0	High
Attia et al. (2017)	1	0	1	1	1	1	1	0	1	1	High
Bastida Castillo et al. (2017)	1	0	1	1	1	0	1	1	1	1	High
Bond et al. (2017)	1	1	0	1	1	1	1	1	1	1	High
Bond et al. (2017b)	1	1	1	1	1	1	1	1	1	1	High
Cronin and Templeton (2008)	1	0	1	1	1	1	1	0	1	0	Low
Enoksen et al. (2009)	1	0	1	1	1	1	1	0	1	0	Low
García-López et al. (2012)	1	0	1	1	1	1	1	1	0	0	Low
García-López et al. (2013)	1	1	1	1	1	1	1	0	0	1	High
Glatthorn et al. (2011)	1	0	1	1	1	1	1	0	0	1	Low
Hanley and Tucker (2019)	1	0	1	1	1	1	1	0	1	0	Low
Haugen et al. (2014b)	1	0	1	1	1	1	1	1	1	0	High
Heredia-Jimenez and Orantes-Gonzalez (2020)	1	1	1	1	1	1	1	1	0	1	High
Viitasalo et al. (1997)	1	0	0	1	1	1	1	0	0	0	Low
Yeadon et al. (1999)	1	0	1	1	1	1	1	0	0	1	Low

**Note:** Provide in the abstract an informative and balanced summary of what was done and what was found (item 1); state specific objectives, including any prespecified hypotheses (item 2); define the eligibility criteria, and the sources and methods of selection of participants (item 3); for each variable of interest, provide sources of data and details of methods of assessment (measurement). Describe comparability of assessment methods if there is more than one group (item 4); explain how quantitative variables were handled in analyses. If applicable, describe which groupings were chosen and why (item 5); provide characteristics of study participants (item 6); summarize key results with reference to study objectives (item 7); discuss limitations of the study, considering sources of potential bias or imprecision. Discuss both direction and magnitude of any potential bias (item 8); give a cautious overall interpretation of results considering objectives, limitations, multiplicity of analyses, results from similar studies, and other relevant evidence (item 9); give the source of funding and the role of the funders for the present study and, if applicable, for the original study on which the present article was based (item 10).

### Effect Measures

The data validity was interpreted according to the concordance value of the data collected by the photocell measurement systems compared to the gold standard (Hopkins et al., 2009). For this purpose, the intraclass correlation coefficient (ICC) was assessed according to the following scale of values: <0.50 (poor), 0.5–0.75 (moderate), 0.75–0.9 (good) and >0.90 (excellent) (Koo and Li, 2016), as well as Pearson's correlation coefficient (r) analysis was conducted with the interpretation as follows: non-significant (r < 0.10), low (0.10–0.30), moderate (0.30–0.50), high (0.50–0.70), very high (0.70–0.90), almost perfect (r > 0.90) and perfect (r = 1.00) (Hopkins et al., 2009).

## Results

### Identification and Selection of Studies

**Figure 1:**
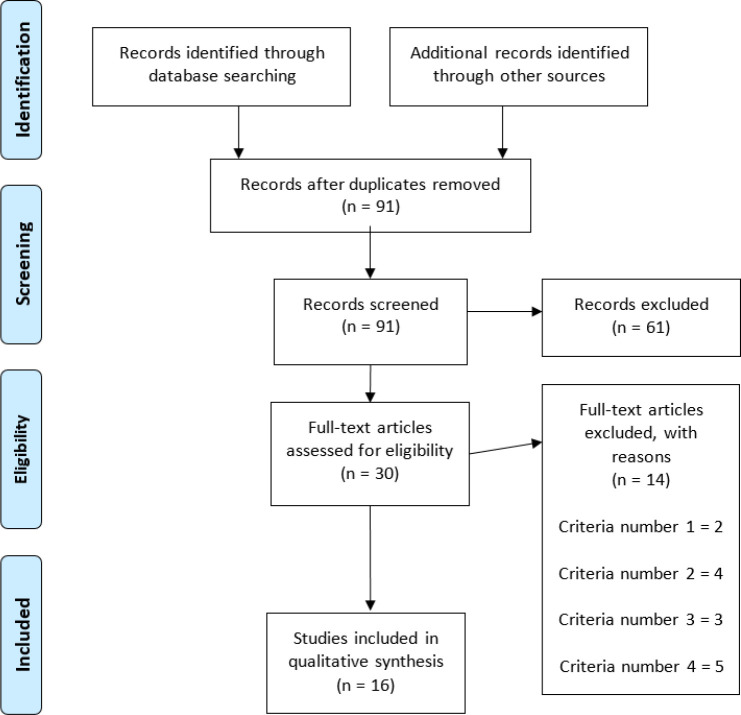
PRISMA flow diagram.

The database search identified a total of 164 titles (PubMed = 61; Web of Science = 48 and, SPORTDiscus = 55). These studies were then exported to reference manager software (EndNote^TM^ X9, Clarivate Analytics, Philadelphia, PA, USA). Duplicates (73 references) were subsequently removed either automatically or manually. The remaining 91 articles were screened for their relevance based on titles and abstracts, resulting in the removal of further 61 studies. Following the screening procedure, 30 articles were selected for in-depth reading and analysis. After reading full texts, further 14 studies were excluded due to not meeting the eligibility criteria (Figure 1).

### Methodological Quality

The overall methodological quality of the cross-sectional studies can be found in Table 2.

### Study Characteristics

Tables 3–5 summarize the characteristics of the studies, the validity of photocells for assessing sports movements and the reliability of photocells for assessing sports movements, respectively.

**Table 3a:** Characteristics of included studies.

Study	Sport		OT			TV TR			DT			PT
Horizontal displacement measurement photocells												
Altmann et al. (2018a)		Team sports		Sprint time	Yes		No	TAG Heuer (La-Chaux-de-Fonds, Switzerland)			Single-beam	
Altmann et al. (2018b)		Team sports		Sprint time	Yes		No	TAG Heuer (La-Chaux-de-Fonds, Switzerland)			Single-beam	
Bastida Castillo et al. (2017)		Athletics		Run time	Yes		No	Chronojump (infrared cell) + WIMU PRO (RealTrack Systems)			100 and 1000 Hz sample rate; Post-processing (S PRO software) Single-beam	
Bond et al. (2017)		Hockey		Sprint time	No		Yes	TCi Photogate (Brower Timing System LLC, Draper, UT, USA)			Laser photocell Single-beam	
Bond et al. (2017b)		Ice hockey		Sprint time	No		Yes	TCi Photogate (Brower Timing System LLC, Draper, UT, USA)			Laser photocell Single-beam	
Cronin and Templeton (2008)		Athletics		Sprint time	No		Yes	Swift Performance Equipment (Lismore, Australia)			Dual-beam (Accuracy 0.01 s)	
García-López et al. (2012)		Physically active		Sprint time	No		Yes	DSD Laser System (DSD Inc., León, Spain)			Single-beam (laser light) Dual-beam (laser light) Sport SPEED-v2.0 (500 Hz) software	
Haugen et al. (2014b)		Athletics		Sprint time	No		Yes	Brower Timing System LLC, (Draper, UT, USA) Biomekanikk AS (Oslo, Norway)			Single-beam (Accuracy 0.01 s) Dual-beam (Accuracy 0.01 s)	
Yeadon et al. (1999)		Physically active		Running speed	Yes		No	Not specified			Single-beam Dual-beam	

**Note**: Hz: hertz; v: version. Table headers: OT (Outcome tested); TV (Test Validity); TR (Test Reliability); DT (Device/Trademark); PT (Photocell Technology).

**Table 3b:** Characteristics of included studies.

Study		Sport	OT			TV			TR	DT		PT
Vertical displacement measurement photocells												
Attia et al. (2017)	Physically active			Jump height	Yes		Yes	Optojump (Microgate, Bolzano, Italy)			1000 Hz sampling rate; Post-processing (Microgate v.3.01.0001) 32 light-emitting diodes	
García-López et al. (2013)	Athletics			Jump height	Yes		Yes	Study 1: SportJump System Pro (DSD Inc., León, Spain) ErgoJump Plus (Bosco System, Byomedic SCP, Barcelona, Spain)			Photocell mat, laser rays, 1000 Hz sample rate (SportJump v1.0 software). Photocell mat, infrared rays, 1000 Hz sample rate. 96 infrared leds	
García-López et al. (2012)	Athletics			Jump height	No		Yes	Study 2: SportJump System Pro (DSD Inc., León, Spain)			Photocell mat	
Glatthorn et al. (2011)	Physically active			Jump height	Yes		Yes	Optojump (Microgate, Bolzano, Italy)			1000 Hz sample rate (Optojump, v. 3.01.0001 software)	
Hanley and Tucker (2019)	Racewalking			Flight and contact time	No		Yes	OptoJump Next (Microgate, Bolzano, Italy)			1000 Hz sample rate (5 baseline LED settings)	
Heredia-Jimenez and Orantes-Gonzalez (2020)	Physically active			Jump height	Yes		No	Sport Jump System Pro (DSD Inc., León, Spain)			Laser rays, 1000 Hz sample rate (SportJump v 2.0 software)	
Viitasalo et al. (1997)	Sprint and marathon			Contact time	No		Yes	Photocell contact mat (no trademark provided)			3000 Hz sample rate	
**Horizontal and vertical displacement measurement photocells**												
Enoksen et al. (2009)	Soccer			Sprint time	Yes		Yes	Photocells and mat Newtest Powertimer 300-series (Newtest Oy, Finland)			−	

**Note:** Hz: hertz; v: version. Table headers: OT (Outcome tested); TV (Test Validity); TR (Test Reliability); DT (Device/Trademark); PT (Photocell Technology).

**Table 3c:** Characteristics of included studies.

Study	GS	PC	Experimental protocol		
			Test	Location of photocells	
				D	H
Horizontal displacement measurement photocells					
Altmann et al. (2018a)	High speed video cameras (100 Hz sampling rate)	15 males (Age: 24.3 ± 1.8 yr.; 178.5 ± 7.4 cm; 74.6 ± 8.7 kg)	3 x 30 m sprint	5, 10 and 30 m System 1: 0.3 m System 2: 0.3 m	1 m 0.64 m (knee) 0.25 m (ankle)
Altmann et al. (2018b)	High speed video cameras (100 Hz sampling rate)	15 males (Age: 24.3 ± 1.8 yr.; 178.5 ± 7.4 cm; 74.6 ± 8.7 kg)	3 x 20 m flying sprint (10–30 m)	10 and 30 m	System 1: 0.64 m (knee) System 2: 1 m (hip)
Bastida Castillo et al. (2017)	Photocell (Chronojump software)	3 males (−)	x6 attempts of 100 Hz (x3) and 1000 Hz (x3) 20 m and 400 m at maximum speed 150 m at different speeds	0, 20, 150 m and 400 m	−
Bond et al. (2017)	3D motion capture (240 Hz sampling rate)	15 males (Age: 18.9 ± 0.7 yr.; 183 ± 7 cm; 86.5 ± 4.7 kg)	5 x 18 m sprint Start at 0.3 m from the starting line.	0, 9 and 18 m	0.99 m
Bond et al. (2017b)	−	17 males (Age: 19.0 ± 0.7 yr.; 184 ± 4 cm; 86.3 ± 6.4 kg)	5 x 9.15 m sprint Start at 0.3 m from the starting line	0 and 9,15 m	0.99 m
(Cronin and Templeton, 2008)	−	9 males and 6 females (Age: 22.7 ± 3.6 yr.; 172 ± 9 cm, 71.8 ± 12.2 kg)	6 x 20 m sprint Start at 0.3 m from the starting line.	10 or 20 m (x3 repetitions each time)	System 1: 0.60 m System 2: 0.80 m
García-López et al. (2012)	−	25 males (Age: 20.5 ± 0.5 yr.; 178 ± 2 cm; 77.5 ± 1.8 kg)	3 x 15 m sprint Start with three supports on the ground: 2 feet and one hand. 3 x 15 m flying sprint (20 m pre−acceleration)	0, 5, 10 and 15m	0.90 (hip), 1.10 m and 1.30 m in each distance
Haugen et al. (2014b)	−	10 males and 15 females (Age: 19 ± 1 yr.; 174 ± 8 cm; 67 ± 10 kg)	2 x 40 m sprint; standing stationary position start	0.5, 20 and 40 m	Single-beam: 1 m. Dual-beam: 1.1 and 1.3 m at the start, 1.3 and 1.5 m at 20 and 40 m
Yeadon et al. (1999)	Panasonic F−15 (sVHS) camera at 50 Hz sampling rate Sony Handycam Pro (Hi−8) camera at 50 Hz sampling rate	1 healthy and physically fit male athlete (Age: unspecified; 190 cm; 80.7 kg)	5 x 9 m at 5, 6, 7, 8 and 9 m·s^−1^	1.6, 1.8, 2.0, 2.2 and 2.4 m	Single-beam: 1.05 m Dual-beam: 1.05 and 1.25 m

Note: cm: centimeters; Hz: hertz; kg: kilograms; m: meters. Table headers: GS (Gold Standard); PC (Population characteristics); D (Distance); H (Height).

**Table 3d:** Characteristics of included studies.

Study	GS	PC	Experimental protocol		
			Test	Location of photocells	
				D	H
Vertical displacement measurement photocells					
Attia et al. (2017)	Force plate Quattro-Jump (500 Hz sample rate)	20 males (Age: 22.50 ± 1.24 yr.; 177.05 ± 7.04 cm; 75.77 ± 13.22 kg)	3−5 attempts SJ, CMJ and CMJ+	−	−
García-López et al. (2013)	Force plate (1000 Hz sample rate)	62 males and 27 females (Age: 20.5 ± 1.4 yr.; 170.5 ± 4.7 cm; 67.8 ± 5.6 kg)	x3 CMJ	−	Ground level
García-López et al. (2012)	−	63 males and 19 females (Age: 20 ± 1.6 yr.; 170.9 ± 5.8 cm; 64.3 ± 7.5 kg)	x3 CMJ	−	Ground level
Glatthorn et al. (2011)	Force plate Quattro-Jump (500 Hz sample rate)	Validity: 20 males (Age: 22 ± 2 yr.; 180 ± 9 cm; 75 ± 10 kg) Reliability: 20 males (Age: 30 ± 5 yr.; 175 ± 10 cm; 68 ± 14 kg)	x3 attempts of SJ, CMJ and CMJ+	−	Ground level
Hanley and Tucker. 2019)	Force plate Kistler (1000 Hz sample rate)	11 males and 7 females (Age: 25.8 ± 4.1 yr.; 172 ± 8 cm; 60.5 ± 7.8 kg)	Running on treadmill and on the ground (11, 12, 13, 14 and 15 km/h in men; −1 km/h in each phase for women)	−	−
Heredia-Jimenez and Orantes-Gonzalez (2020)	Force plate Kistler (200 Hz sample rate)	20 males (Age: 23.4 ± 2.9 yr.; 179 ± 6 cm; 75.2 ± 9.4 kg)	x2 CMJ	−	Ground level
Viitasalo et al. (1997)	Force plate Kistler (400 Hz sample rate) and force plate TR Testy Oy (170 Hz sample rate)	2 males (1 sprinter and 1 marathoner)	4–6 repetitions of running at 4, 5,5 km/h and at maximum speed	−	10, 23, 37 and 46 mm
**Horizontal and vertical displacement measurement photocells**					
Enoksen et al. (2009)	Force plate (1000 Hz) + Dual-beam photocells	20 males (Age: 19.1 ± 3.5 yr.; 179 ± 8 cm; 72.6 ± 7.8 kg)	x3 CMJ and SJ (Photocell mat)	−	−
			3 x 40 m sprint Standing start	20 and 40 m	-

Note: cm: centimeters; CMJ: Countermovement jump with arms on hips; CMJ+: Countermovement jump with arm swing; Hz: hertz; kg: kilograms; km/h: kilometers per hour; m: meters; mm: millimeters;

SJ: Squat jump. Table headers: GS (Gold Standard); PC (Population characteristics);

D (Distance); H (Height).

**Table 4a:** Validity of photocells for assessing sports movements.

Study	Device	Exercise	Measured variable	ICC	r	LoA (95%)	Lessons learned and concluding remarks
Horizontal displacement measurement photocells							
Altmann et al. (2018a)	TAG Heuer	Standing start sprint (30 m)	0.64 m height, time at: 5 m 10 m 30 m 1 m height, time at: 5 m 10 m 30 m	0.134 0.278 0.657 0.008 0.400 0.869	0.351** 0.597** 0.848** 0.449 0.904 0.905*	−0.267–0.089 s −0.280–0.080 s −0.276–0.066 s 0.037–0.193 s 0.038–0.128 s 0.006–0.120 s	Questionable validity at 5 and 10 m Acceptable validity in 30 m, especially at 1 m height Data not interchangeable at different heights
Altmann et al. (2018b)	TAG Heuer	Flying start sprint (20 m)	0.64 m height 1 m height	0.978 0.969	0.985 0.991*	−0.060–0.120 m/s −0.013–0.121 m/s	Device valid at both heights for a 20 m sprint with a flying start
Bastida Castillo et al. (2017)	Chronojump + WIMU PRO	Standing start maximal runs (20 and 400 m), and a 150 m simulated circuit	At 100 Hz sample rate: 20 m 150 m 400 m At 1000 Hz sample rate: 20 m 150 m 400 m	1.000 1.000 1.000	1.000** 1.000** 1.000**		Integration of the two devices obtained valid results
				1.000 1.000 1.000	1.000** 1.000** 1.000**		

Note: Hz: hertz; ICC: Intraclass correlation coefficient; LoA: Limit of agreement; m: meters; ms: milliseconds; r: Pearson's coefficient; s: seconds. *Significant differences with respect to the gold standard: * p < 0.01, ** p < 0.001

**Table 4b:** Validity of photocells for assessing sports movements.

Study	Device	Exercise	Measured variable	ICC	r	LoA (95%)	Lessons learned and concluding remarks
Vertical displacement measurement photocells							
Attia et al. (2017)	Optojump	Squat jump CMJ CMJ+	Jump height	0.989 0.994 0.982	0.978** 0.990** 0.968**	−12.29; −11,04 cm −11.56; −10.61 cm −5.74; −13.25 cm	Valid device but not interchangeable with a force plate
García-López et al. (2013)	SportJump System Pro ErgoJump Plus	CMJ	Flight time Jump height	0.95−0.97		10.4–10.9 ms 0.013–0.015 m 45.1–56.5 ms 0.052–0.065 m	SportJump System Pro was a valid device. Ergojump Plus obtained questionable validity. None of the devices is interchangeable with a force plate
			Flight time Jump height	0.45−0.57			
Glatthorn et al. (2011)	OptoJump	Squat jump CMJ CMJ+	Jump height	0.997 0.998 0.998		**Systematic bias** 0.9 cm 1.0 cm 1.3 cm	Valid device
Heredia-Jimenez and Orantes-Gonzalez (2020)	Sport Jump System Pro	CMJ	Flight time Numerical integration	0.960 0.82		0.8–2.9 cm −6–10 cm	Valid device
**Horizontal and vertical displacement measurement photocells**							
Enoksen et al. (2009)	Newtest Powertimer 300−series	Squat jump CMJ	Jump height Jump height 0–20 m time 20–40 m time 0–40 m time		0.65* 0.75**	−3.4–6.8 cm −2.4–8.0 cm	Device validity could not be confirmed
					0.33 0.34 0.17**	0.3%–2.1% −0.04–0.05 s −0.02–0.10 s	
		Sprint (40 m)					

Note: cm: centimeters; CMJ: Countermovement jump with arms on hips; CMJ+: Countermovement jump with swing arms; ICC: Intraclass correlation coefficient; LoA: Limit of agreement; m: meters; ms: milliseconds; r: Pearson's coefficient; s: seconds.

*Significant differences with respect to the gold standard: *p < 0.01, ** p < 0.001

**Table 5a:** Reliability of photocells for assessing sports movements.

Study	Device	Reliability type	Measured variable	ICC	CV	Measurement errors	Lessons learned and concluding remarks by authors
Horizontal displacement measurement photocells							
Bond et al. (2017)	TC Photogate	Intra-session and intra-device	Sprint time: 0–3 m 3–6 m 6–9 m 0–9 m 0–18 m			**TE:** 0.03 s 0.03 s 0.01 s 0.05 s 0.05 s	Reliable system, but sensitive to factors such as body tilt, body segments, and device height.
Bond et al. (2017b)	TC Photogate	Intra-session and intra-device	Sprint time			**TE:** 0.03–0.06 s	Reliable system TE lowers as the number of repetitions performed increased
Cronin and Templeton (2008)	Dual beam (Swift Performance Equipment, Lismore, Australia)	Intra-session and intra-device	Sprint time (0.60 m height): 10 m time 20 m time Sprint time (0.80 m height): 10 m time 20 m time		1.1 % 0.69 % 1.2 % 0.83 %		Reliable device at both heights and distances
García-López et al. (2012)	DSD Laser System	Intra-session and intra-device	Sprint time (SB): 5 m 10 m 15 m Sprint time (DB): 5 m 10 m 15 m Flying sprint time (SB): 5 m 10 m 15 m Flying sprint time (DB): 5 m 10 m 15 m	0.71 0.80 0.89 0.84 0.89 0.95 0.53 0.71 0.81 0.74 0.87 0.93	1.58 ± 1.83 % 1.07 ± 1.10 % 0.88 ± 0.77 % 1.29 ± 1.58 % 0.95 ± 0.95 % 0.71 ± 0.63 % 1.69 ± 1.56 % 1.51 ± 1.12 % 1.33 ± 0.85 % 1.48 ± 1.09 % 0.85 ± 0.74 % 0.74 ± 0.47 %		Reliable SB at all distances and races, especially from 10 m in acceleration races and 15 m in flying sprints Reliable DB at all distances and races, especially from 5 m in acceleration races and 10 m in flying sprints
Haugen et al. (2014b)	Brower Timing System Biomekanikk	Intra-session and intra-device	Sprint time SB vs DB: 0–20 m 20–40 m		1.4 % 1.2 %	**SEM** 0.02 s 0.02 s	

Note: CV: Coefficient of variation; DB: Dual beam; ICC: Intraclass correlation coefficient; m: meters; s: seconds; SB: Single beam; SEM: Standard error of measurement; TE: Typical error.

**Table 5b:** Reliability of photocells for assessing sports movements.

Study	Device	Reliability type	Measured variable	ICC	CV	Measurement errors	Lessons learned and concluding remarks by authors
Vertical displacement measurement photocells							
Attia et al. (2017)	Optojump	Intra-session and intra-device	Jump height (SJ) Jump height (CMJ) Jump height (CMJ+)	0.980 0.992 0.999	6.47 % 3.70 % 1.76 %	**SEM:** 1.16 cm 0.79 cm 0.45 cm	Highly reliable device in the three exercises evaluated
García-López et al. (2013)	SportJump System Pro ErgoJump Plus	Intra-session and intra-device	Flight time (CMJ) Jump height (CMJ) Flight time (CMJ) Jump height (CMJ)		1.21 ± 0.81 % 2.98 ± 2.01 % 6.61 ± 4.81 % 15.94 ± 11.48 %		SportJump System Pro is a reliable device ErgpJump Plus showed questionable reliability
	SportJump System Pro	Intra-session and intra-device	Flight time (CMJ) Jump height (CMJ)		1.14 ± 0.56 % 2.28 ± 1.13 %		Reliable device
Glatthorn et al. (2011)	Optojump	Inter-session and intra-device	Jump height (SJ) Jump height (CMJ) Jump height (CMJ+)	0.982 0.989 0.984	3.1 % 2.2 % 2.8 %	**Systematic bias** −0.32 cm −0.11 cm 0.36 cm	Reliable device
Hanley and Tucker (2019)	OptoJump Next	Intra-session and intra-device	Contact time (RW): Treadmill Overground Flight time (RW): Treadmill Overground	0.599–0.968 0.552– 0.984 0.311– 0.934 0.867– 0.995		**Systematic bias** −0.04–0.042 s −0.011–0.024 s −0.042–0.04 s −0.024–0.010 s	Reliable device, especially the 2−2 configuration on treadmill and the 0−0 configuration on the surface
Viitasalo et al. (1997)	Photocell contact mat	Intra-session and intra-device	Contact time (sprint−marathon: 10 mm height 23 mm height 37 mm height 46 mm height		1.25–- 1.64 % 2.49–- 1.42 % 2.67–- 2.53 % 2.33–- 2.97 %		Reliable device, especially when placed 10 mm above the ground
**Horizontal and vertical displacement measurement photocells**							
Enoksen et al. (2009)	Newtest Powertimer 300-series	Intra−session and intra−device	Jump height (SJ) Jump height (CMJ) 0–20 m sprint time 0–40 m sprint time		0.7 % 0.2 % 0.4 % 0.4 %		Reliable device

Note: cm: centimeters; CMJ: Countermovement jump with arms on hips; CMJ+: Countermovement jump with swing arms; CV: Coefficient of variation; ICC: Intraclass correlation coefficient; m: meters; mm: millimeters; RW: Racewalking; s: seconds; SEM: Standard error of measurement; SJ: Squat jump.

## Discussion

Photocells have been widely used in sports to measure performance in both vertical and horizontal displacements (Haugen and Buchheit, 2016; Viitasalo et al., 1997). However, it is essential to ensure that the tools used are valid and reliable, to allow an objective interpretation of performance changes. Therefore, the aim of the present systematic review was to summarize and analyze the concurrent validity and reliability of photocells in sports, specifically when assessing horizontal displacements (i.e., sprinting time and velocity) and vertical displacements (i.e., flight time and jump height). The main findings revealed that quantifying the timing of vertical displacement is valid and reliable, whilst assessing horizontal displacements in an appropriate manner is a delicate task, especially when dealing with extremely small-time intervals such as in the first meters of a sprint. Nevertheless, the majority of articles indexed in this review have not reported the adjustment of the sensitivity of the infrared sensor, which controls the trigger sensitivity of the signal (Altmann et al., 2018a, 2018b; Haugen et al., 2014a; Haugen and Buchheit, 2016).

### Factors Affecting the Validity and Reliability of Photocells for Monitoring Horizontal Displacement

It is well known that during sprinting the movement of the arms and legs is wider and faster than that of the trunk and the total center of mass, which might cause the timing gate to be prematurely or repeatedly triggered when using single beam photocells (Doma et al., 2023; Haugen and Buchheit, 2016). Altmann et al. (2018) showed that measuring horizontal displacement speed from a standing starting position with single beam photocells yielded a poor ICC (ICC = 0.278) in the first 10 m, likely because the lower running speeds and shorter time frames during the acceleration phase increased the magnitude of this error (Altmann et al., 2018; García-López et al., 2012). When starting from a crouched position, another source of error in the first meters is added because the body tends to tilt forward at least until the 13^th^ step (or approximately 18.5 m) (Nagahara et al., 2014) which would also trigger the photocell beam before the center of mass reaches that position. For sprints longer than 20 m or with a flying start, validity of single beam photocells has been proven excellent (Altmann et al., 2018; Bastida Castillo et al., 2017). Another factor to consider is the photocell height, for instance, they can be placed near the ground to measure the start in standing position starting sprints (Altmann et al., 2018b) or at a height varying from knee to head height to detect the runner breaking the beam. This height can be adjusted to match specific needs (i.e., different athletes’ heights, different starting positions etc.). Photocells at lower beam heights (< 0.64 m) correspond to the shortest times, due to the fact that on many occasions the light beam is cut by the thigh, being sometimes difficult to detect by dual-beam or split-beam and post-processing systems. However, it has been shown that there were no significant differences between 0.64 and 1 m single beam photocell heights for a 20-m flying start sprint, decreasing the relative error (Altmann et al., 2018b), but there were differences with a split start (standing with one foot in front of the other) (Altmann et al., 2018a). This discrepancy is attributed to the fact that, for an initial distance of 0.30 m from the first timing gate, the ankle of the back foot could pass the initial timing gate simultaneously with the hip. Therefore, placing initial timing gates below the knee could improve their accuracy (Altmann et al., 2018b).

As it has been observed, during the acceleration phase over the first 10 m, the way of starting (standing or 3-point or 4-point) is a decisive factor, and the utmost rigor should be applied when deciding on the type of start to use as they are not comparable and cannot be used interchangeably (Haugen and Buchheit, 2016). To avoid these issues, the use of dual-beam or post-processing tools has been suggested (Altmann et al., 2018b), although the sensitivity of the infrared receiver should also be considered, providing further evidence of the superior validity of dual-beam systems compared to single-beam systems when aiming to avoid false triggering produced by swinging arms or legs (Haugen and Buchheit, 2016). This factor is not generally reported, but in some measuring devices it can be adjusted so that the light beam can identify small segments such as fingers, arms or the trunk, in order to consider only the segment of interest and avoid false triggering. However, 0.60 m height dual-beam photocells produce significantly faster times than 0.80-m height photocells for 10- and 20-m standing start sprints (Cronin and Templeton, 2008). Cronin and Templeton (2008) did not report whether these heights corresponded to the upper or the lower photocell, nor the distance between the two beams. Surprisingly, we found no investigations that aimed to assess the ideal distance between both beams in a dual-beam system, although there seems to be a consensus to place them 0.2 m apart. The results show that the relative error decreases considerably from 30 m onwards, likely because the photocell beam is no longer triggered early by the forward-leaning trunk as it becomes upright after just 16 steps from a block start (Nagahara et al., 2014).

### Factors Affecting the Validity and Reliability of Photocells for Monitoring Vertical Displacement

Similarly as for horizontal displacements, photocell systems have been used for vertical displacement measurement, and were generally compared to force plates with sampling rates between 500 and 1000 Hz (Attia et al., 2017; Cronin and Templeton, 2008; García-López et al., 2013; Hanley and Tucker, 2019). The tests used were a squat jump (SJ), a counter-movement jump (CMJ) and a loaded counter-movement jump (CMJ+). The inter-trial intraclass correlation coefficient (ICC) of SJ, CMJ and CMJ+ heights obtained in the studies included in this review (range: 0.93 to 0.99) was in agreement (ICC: 0.95) with that reported by Nuzzo et al. (2011) measured with a Myotest accelerometer. Furthermore, if data are homoscedastic as in the study by Attia et al. (2017), SEM analyses may be more useful to establish absolute reliability (Atkinson and Nevill, 1998). In heteroscedastic data analyses including the coefficient of variation (CV) are recommended (Atkinson and Nevill, 1998). These results obtained from different manuscripts suggest that photocells are a reliable instrument for measuring vertical jump height estimated from flight time when compared to the gold standard (force plates).

Regarding validity, the different indexed studies (Attia et al., 2017; García-López et al., 2013; Glatthorn et al., 2011; Heredia-Jimenez and Orantes-Gonzalez, 2020) show a certain degree of agreement that photocells are valid systems for measuring vertical displacement when compared to force plates (gold standard). Despite ICCs for validity very close to 1 in all manuscripts, it is important to point out that it is a system that presents a certain systematic error of underestimation (Attia et al., 2017; García-López et al., 2013; Glatthorn et al., 2011) in jump height recorded by photocells. These findings could be attributed to the number of LEDs (32 LEDs), an adequate sampling frequency (1000 Hz), as well as the height of the LED barrier with respect to the ground, because the higher the photocells are, the lower the measured flight time will be. For example, for a gravitational value of 9.81 m·s^−2^ and a jump of 0.5 s, the jump distance can be calculated as 30.656 cm. Photocells with a small variation of 1 cm in height with respect to the ground would measure a time of flight of 0.49191 s, thus giving a jump height of 29.672 cm (i.e., 0.98 cm less). Despite these significant underestimates of the jump height data recorded by photocells, these systems appear to be valid and reliable, since there were significant correlations and systematic errors among the methods, and it was possible to establish prediction equations to overcome the underestimates shown by photocells versus force plates.

## Conclusions

Based on this review, the method aiming at assessing vertical jump height through flight time with photocells appears to show a strong agreement with force plates (gold standard), yet is not interchangeable. The risk of collecting confusing data leads to misinterpretations that can affect the quality of training, and therefore athletes’ health and performance; therefore, coaches and trainers should be cautious when selecting the measuring instrument to assess and monitor athletes’ jump performance. Regarding the validity and reliability of photocells for monitoring horizontal displacement, it seems that double-beam systems, compared to single-beam systems, are more valid and reliable when it comes to avoiding false triggers caused by swinging arms or legs. This difference is particularly noticeable in the acceleration phase (first 10 m), in which the starting position (flying start, standing start) presents a marked relevance.

## References

[ref1] Altmann, S., Ringhof, S., Becker, B., Woll, A., & Neumann, R. (2018a). Error-correction processing in timing lights for measuring sprint performance: Does it work?. International Journal of Sports Physiology and Performance, 13(10), 1400–1402. 10.1123/ijspp.2017-0596. 29809060

[ref2] Altmann, S., Spielmann, M., Engel, F. A., Neumann, R., Ringhof, S., Oriwol, D., & Haertel, S. (2018b). Validity of Single-Beam Timing Lights at Different Heights. Journal of Strength and Conditioning Research, 31(7), 1994–1999. 10.1519/JSC.0000000000001889. 28277431

[ref3] Atkinson, G., & Nevill, A. M. (1998). Statistical methods for assessing measurement error (reliability) in variables relevant to sports medicine. Sports Medicine (Auckland, N.Z.), 26(4), 217–238. 10.2165/00007256-199826040-00002. 9820922

[ref4] Attia, A., Dhahbi, W., Chaouachi, A., Padulo, J., Wong, D., & Chamari, K. (2017). Measurement errors when estimating the vertical jump height with flight time using photocell devices: The example of Optojump. Biology of Sport, 1, 63–70. 10.5114/biolsport.2017.63735. PMC5377563 28416900

[ref5] Bastida Castillo, A., Gómez Carmona, C. D., Pino Ortega, J., & de La Cruz Sánchez, E. (2017). Validity of an inertial system to measure sprint time and sport task time: A proposal for the integration of photocells in an inertial system. International Journal of Performance Analysis in Sport, 17(4), 600–608. 10.1080/24748668.2017.1374633.

[ref6] Bataller-Cervero, A. V., Gutierrez, H., DeRentería, J., Piedrafita, E., Marcén, N., Valero-Campo, C., Lapuente, M., & Berzosa, C. (2019). Validity and Reliability of a 10 Hz GPS for Assessing Variable and Mean Running Speed. Journal of Human Kinetics, 67, 17–24. 31523303 10.2478/hukin-2018-0084 PMC6714352

[ref7] Bond, C. W. ., Willaert, E. M., & Noonan, B. C. (2017). Comparison of Three Timing Systems: Reliability and Best Practice Recommendations in Timing Short-Duration Sprints. Journal of Strength and Conditioning Research, 31, 1062–1071. 27398914 10.1519/JSC.0000000000001566

[ref8] Bond, C. W., Willaert, E. M., Rudningen, K. E., & Noonan, B. C. (2017b). Reliability of Three Timing Systems Used to Time Short on Ice-Skating Sprints in Ice Hockey Players. Journal of Strength and Conditioning Research, 31(12), 3279–3286. 10.1519/JSC.0000000000002218. 28858060

[ref9] Cronin, J. B., & Templeton, R. L. (2008). Timing Light Height Affects Sprint Times. Journal of Strength and Conditioning Research, 22(1), 318–320. 10.1519/JSC.0b013e31815fa3d3. 18296992

[ref10] Doma, K., Connor, J. D., Nakamura, F. Y., & Leicht, A. S. (2023). Intra-Session Reliability of Sprint Performance on a Non-Motorised Treadmill for Healthy Active Males and Females. Journal of Human Kinetics, 87, 163–171. 10.5114/jhk/163180 37559768 PMC10407314

[ref11] Enoksen, E., Tønnessen, E., & Shalfawi, S. (2009). Validity and reliability of the Newtest Powertimer 300-series® testing system. Journal of Sports Sciences, 27(1), 77–84. 10.1080/02640410802448723. 19031330

[ref12] Faude, O., Koch, T., & Meyer, T. (2012). Straight sprinting is the most frequent action in goal situations in professional football. Journal of Sports Sciences, 30(7), 625–631. 10.1080/02640414.2012.665940. 22394328

[ref13] García-López, J., Morante, J. C., Ogueta-Alday, A. C., González-Lázaro, J., Rodríguez-Marroyo, J. A., & Villa, G. (2012). The use of single and double beam photocells for speed measurement in racing: DSD Laser System ®. RICYDE: Revista Internacional de Ciencias del Deporte, 8(30), 324–333. 10.5232/ricyde2012.03003.

[ref14] García-López, J., Morante, J. C., Ogueta-Alday, A., & Rodríguez-Marroyo, J. A. (2013). The type of mat (Contact vs. Photocell) affects vertical jump height estimated from flight time. Journal of Strength and Conditioning Research, 27(4), 1162–1167. 10.1519/JSC.0b013e31826520d7. 22744294

[ref15] Glatthorn, J. F., Gouge, S., Nussbaumer, S., Stauffacher, S., Impellizzeri, F. M., & Maffiuletti, N. A. (2011). Validity and Reliability of Optojump Photoelectric Cells for Estimating Vertical Jump Height. Journal of Strength and Conditioning Research, 25(2), 556–560. 10.1519/JSC.0b013e3181ccb18d. 20647944

[ref16] Hanley, B., & Tucker, C. B. (2019). Reliability of the Optojump next system for measuring temporal values in elite racewalking. Journal of Strength and Conditioning Research, 33(12), 3438–3443. 10.1519/jsc.0000000000003008. 30640307

[ref17] Haugen, T. A., Tønnessen, E., Hisdal, J., & Seiler, S. (2014a). The role and development of sprinting speed in soccer. International Journal of Sports Physiology and Performance, 9(3), 432–441. 10.1123/IJSPP.2013-0121. 23982902

[ref18] Haugen, T. A., Tønnessen, E., & Seiler, S. K. (2012). The Difference Is in the Start: Impact of Timing and Start Procedure on Sprint Running Performance. Journal of Strength and Conditioning Research, 26(2), 473. 10.1519/JSC.0b013e318226030b. 22233797

[ref19] Haugen, T. A., Tønnessen, E., Svendsen, I. S., & Seiler, S. (2014b). Sprint time differences between single-and dual-beam timing systems. Journal of Strength and Conditioning Research, 28(8), 2376–2379. 10.1519/JSC.0000000000000415. 24531428

[ref20] Haugen, T., & Buchheit, M. (2016). Sprint Running Performance Monitoring: Methodological and Practical Considerations. Sports Medicine, 46(5), 641–656. 10.1007/s40279-015-0446-0. 26660758

[ref21] Heredia-Jimenez, J., & Orantes-Gonzalez, E. (2020). Comparison of three different measurement systems to assess the vertical jump height. Revista Brasileira de Medicina do Esporte, 26(2), 143–146. 10.1590/1517-869220202602185305.

[ref22] Hopkins, W. G. (2000). Measures of Reliability in Sports Medicine and Science. Sports Medicine, 30(1), 1–15. 10.2165/00007256-200030010-00001. 10907753

[ref23] Hopkins, W. G., Marshall, S. W., Batterham, A. M., & Hanin, J. (2009). Progressive statistics for studies in sports medicine and exercise science. Medicine and Science in Sports and Exercise, 41(1), 3–13. 10.1249/MSS.0b013e31818cb278. 19092709

[ref24] McBride, J. M., Triplett-McBride, T., Davie, A., & Newton, R. U. (2002). The effect of heavy-vs. Light-load jump squats on the development of strength, power, and speed. Journal of Strength and Conditioning Research, 16(1), 75–82. 11834109

[ref25] Moher, D., Shamseer, L., Clarke, M., Ghersi, D., Liberati, A., Petticrew, M., Shekelle, P., Stewart, L. A., & PRISMA-P Group. (2015). Preferred reporting items for systematic review and meta-analysis protocols (PRISMA-P) 2015 statement. Systematic Reviews, 4, 1. 10.1186/2046-4053-4-1. 25554246 PMC4320440

[ref26] Nagahara, R., Matsubayashi, T., Matsuo, A., & Zushi, K. (2014). Kinematics of transition during human accelerated sprinting. Biology Open, 3(8), 689–699. 10.1242/bio.20148284. 24996923 PMC4133722

[ref27] Nuzzo, J. L., Anning, J. H., & Scharfenberg, J. M. (2011). The reliability of three devices used for measuring vertical jump height. Journal of Strength and Conditioning Research, 25(9), 2580–2590. 10.1519/JSC.0b013e3181fee650. 21804426

[ref28] O’Reilly, M., Caulfield, B., Ward, T., Johnston, W., & Doherty, C. (2018). Wearable Inertial Sensor Systems for Lower Limb Exercise Detection and Evaluation: A Systematic Review. Sports Medicine, 48(5), 1221–1246. 10.1007/s40279-018-0878-4. 29476427

[ref29] Romero-Franco, N., Jiménez-Reyes, P., Castaño-Zambudio, A., Capelo-Ramírez, F., Rodríguez-Juan, J. J., González-Hernández, J., Toscano-Bendala, F. J., Cuadrado-Peñafiel, V., & Balsalobre-Fernández, C. (2017). Sprint performance and mechanical outputs computed with an iPhone app: Comparison with existing reference methods. European Journal of Sport Science, 17(4), 386–392. 10.1080/17461391.2016.1249031. 27806673

[ref30] Soler-López, A., García-de-Alcaraz, A., Moreno-Villanueva, A., & Pino-Ortega, J. (2022). Concurrent Validity and Reliability of Devices to Measure Jump Height in Men’s Handball Players. Sensors (Basel, Switzerland), 22(23), 9070. 10.3390/s22239070. 36501772 PMC9738152

[ref31] Uysal, H. Ş., Ojeda-Aravena, A., Ulaş, M., Martín, E. B., & Ramirez-Campillo, R. (2023). Validity, Reliability, and Sensitivity of Mobile Applications to Assess Change of Direction Speed. Journal of Human Kinetics, 87, 217–228. 37559771 10.5114/jhk/167465 PMC10407321

[ref32] Viitasalo, J. T., Luhtanen, P., Mononen, H. V., Norvapalo, K., Paavolainen, L., & Salonen, M. (1997). Photocell Contact Mat: A New Instrument to Measure Contact and Flight Times in Running. Journal of Applied Biomechanics, 13, 254–266.

[ref33] Willberg, C., Kohler, A., & Zentgraf, K. (2023). Construct Validity and Applicability of a Team-Sport-Specific Change of Direction Test. Journal of Human Kinetics, 85, 115–126. 10.2478/hukin-2022-0115 36643841 PMC9808802

[ref34] Wong, P., Chaouachi, A., Chamari, K., Dellal, A., & Wisloff, U. (2010). Effect of preseason concurrent muscular strength and high-intensity interval training in professional soccer players. Journal of Strength and Conditioning Research, 24(3), 653–660. 10.1519/JSC.0b013e3181aa36a2. 19816215

[ref35] Yeadon, M. R., Kato, T., & Kerwin, D. G. (1999). Measuring running speed using photocells. Journal of Sports Sciences, 17(3), 249–257. 10.1080/026404199366154. 10362392

